# Monitoring of wearing and occlusion times with smart shutter glasses—A proof of concept

**DOI:** 10.1371/journal.pone.0270361

**Published:** 2022-06-24

**Authors:** Frank R. Ihmig, Kai Januschowski, Timo Koch, Thomas Velten, Annekatrin Rickmann

**Affiliations:** 1 Fraunhofer Institute for Biomedical Engineering IBMT, Sulzbach, Saarland, Germany; 2 Eye Clinic Sulzbach, Knappschaft Hospital Saar, Sulzbach, Saarland, Germany; 3 Klaus Heimann Eye Research Institute (KHERI), Knappschaft Hospital Saar, Sulzbach, Germany; Cairo University Kasr Alainy Faculty of Medicine, EGYPT

## Abstract

**Purpose:**

To develop and evaluate an electronic glasses frame for smart liquid crystal shutter glasses that monitors wearing and occlusion times to potentially improve therapy adherence in amblyopia therapy of children.

**Methods:**

The first generation of an electronic glasses frame for adults was further developed, miniaturized and functionally tested in a proof of concept study on a small group of healthy children. Seven healthy children (4 females, 3 males, 2–9 years) were enrolled in the study. The subjects were instructed to wear the smart shutter glasses and to record their activities in daily life. Averaged and individual results were calculated for the precision of wearing position detection and activity recognition. Also, the proper execution of the configured occlusion pattern was observed.

**Results:**

The first generation of an electronic glasses frame for smart liquid crystal shutter glasses in a miniaturized form factor for children. A key element is the implementation of the adaptive shutter operation and of smart algorithms for real-time therapy monitoring. In the proof of concept study, these algorithms monitored the state of wearing position, the wearer’s activity and the configured occlusion pattern. The average agreement of the detected states of wearing position was 72.6%. The average activity recognition match was 77.3%. The removal of the glasses was 100% correctly detected and the occlusion was 100% halted when active motion was recognized.

**Conclusion:**

The assembled smart shutter glasses for children are suitable for demonstrating the feasibility of continuous therapy monitoring by calculating wearing and occlusion times due to smart algorithms for wearing position detection, activity recognition, and occlusion monitoring. However, further research and studies are necessary to optimize the individual fit and performance of this wearable therapeutic device.

## Introduction

One of the most common treatable visual disorders in children is amblyopia [1], which affects their daily life and impacts their future, e.g. their job opportunities [2–6]. A therapy should be started early in the sensitive phase of visual development with e.g. refractive correction and occlusion of the better eye [7, 8]. However, the success of the therapy depends in particular on compliance, i.e. on actual wearing times of the occlusion patch, which is often uncomfortable and disfiguring and leads therefore to a problematic adherence [9]. Furthermore, therapy monitoring is quite difficult. Although thermosensors could monitor compliance, they are still not being used in daily practice [10–14].

To improve this, electronic "shutter glasses" with liquid crystal lenses have been developed. These occlude the better-seeing eye by rhythmically blurring the lens and can be combined with thermosensors [15, 16]. However, even with these glasses, compliance decreases over the course of therapy and resembles patching after three months [17]. This approach is nevertheless promising, as most children require refractive correction and such liquid crystal shutter glasses could effectively replace the occlusion patch in children with mild to moderate amblyopia [18].

For a child-friendly interaction concept with the aims of improving acceptance and increasing compliance, our objective was to go beyond the state of the art by developing sensor-based shutter glasses, that can monitor wearing and occlusion times and adapt the occlusion rhythm to the physical activity state of the individual. In our previous work [19], we developed the first generation of an electronic glasses frame with capacitive sensing of the wearing position, since incorrect positioning can occur unintentionally or intentionally, e.g. to look past the covered lens. In addition, an accelerometer was included in order to detect certain activity patterns. The functionality was evaluated with a group of healthy adults and the results showed robust measurements of activity and wearing position detection [19]. This is the base to realize the continuous therapy monitoring by calculating correct wearing and occlusion times.

In this manuscript, we describe the development and first pilot data of the first generation of an electronic glasses frame that has a miniaturized form factor for children. A key element is the implementation of the adaptive shutter operation and of smart algorithms for real-time therapy monitoring. The aim of this study is to demonstrate that these miniaturized smart liquid crystal shutter glasses can monitor the prescribed wearing and occlusion times on a daily basis, can detect the wearing position in a robust way and can recognize movement patterns of the wearer’s activity in a small group of healthy children.

## Materials and methods

### Miniaturized electronic design of glasses frame

**Fig 1:**
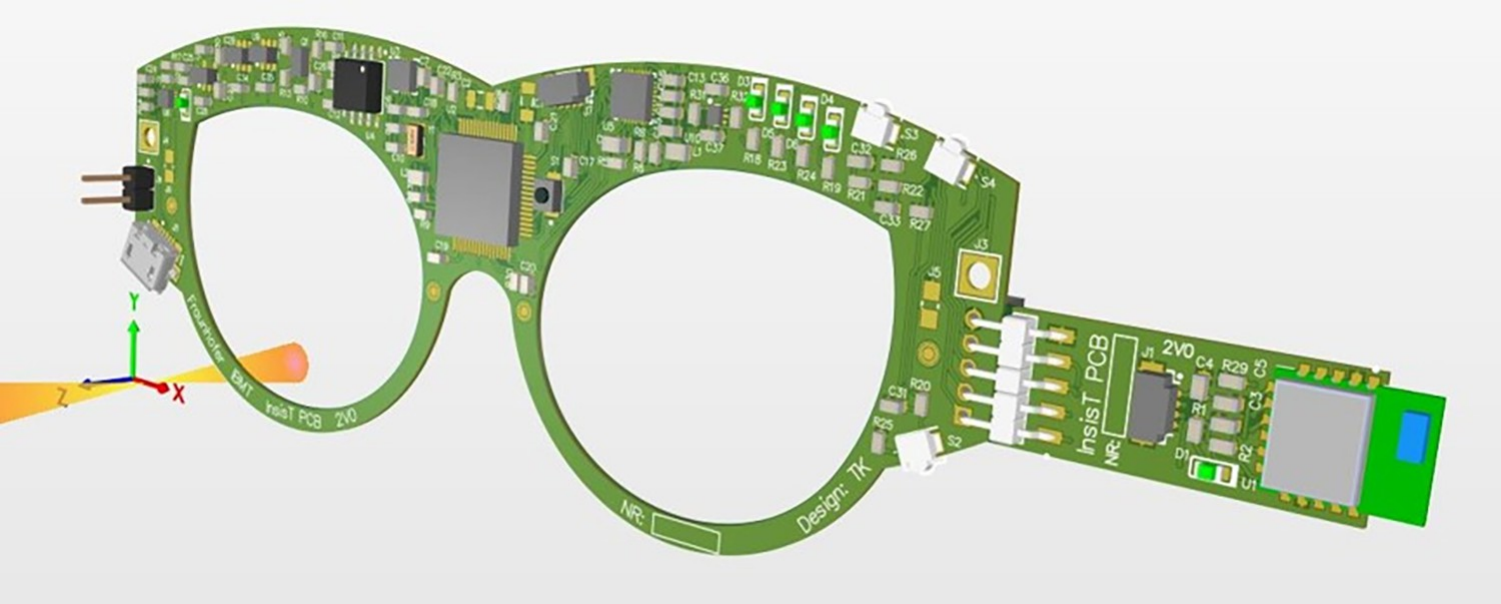
3D model of miniaturized electronic design of glasses frame for children.

The advanced electronic glasses frame for children is based on the same electronic components as the first generation for adults [19] but has a miniaturized form factor (Fig 1).

The miniaturized electronic design was supplemented by customized liquid crystal display (LCD) lenses and its control circuitry. The power management integrated circuit for active shutter 3D glasses TPS65735RSNR (Texas Instruments, Dallas, TX, USA) is used to perform the shutter operation. USB and Bluetooth 4.2 connectivity is available as it was in the first generation. The Bluetooth module is integrated as part of the left temple and the battery is located on the opposite side. USB charging was used as power supply for the lithium-polymer rechargeable battery (3.7 V, 170 mAh).

### Event data acquisition

The flash memory is used to store the therapy events. All events consist of 1 byte of information and there are two types of events: (1) **Therapy events**–this type provides information related to the states for wearing position, activity, and occlusion. An event is stored each time a change occurs during the therapy session (position of the glasses, occlusion of a lens or a different activity detected). (2) **General events**–this type provides information related to the system such as battery state, new therapy session, daily therapy goal achieved, among others. These events have the most significant bit set and the rest of bits are just increased in a consecutive way to list all the events. By doing this, 1 byte is enough to store both types of events.

All the therapy events are stored one after the other as they appear. The reading of the events from the flash memory can be done in multiples of 9 bytes since each event is 9 bytes long: time stamp (4 bytes) + event id (4 bytes) + event data (1 byte).

### Algorithms for real-time therapy monitoring

The essential algorithms to realize the continuous therapy monitoring by calculating wearing and occlusion times are wearing position detection, activity recognition, and occlusion monitoring. They are described in the following:

#### Wearing position detection

The wearing position detection is achieved by using an integrated touch sensing controller peripheral of the microcontroller that has been described in our previous work [19]. Measurement values are obtained based on the contact of the wearer with the conductive temples of the glasses when the therapy session is running on the glasses. The state of wearing position (correct position/wrong position/glasses taken off) is calculated after averaging 32 sensor readings and updated every second. The determined wearing position is essential for the wearing time calculation, since the wearing time is calculated as sum of the durations of all correct position states. The daily therapy goal is achieved when the corresponding timer matches the prescribed wearing time.

**Fig 2:**
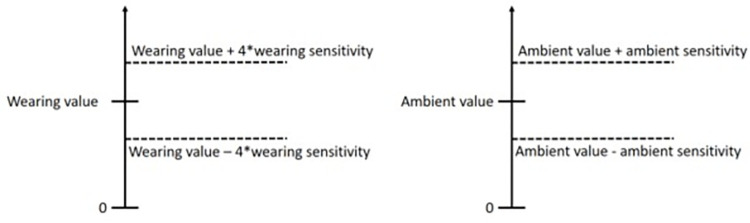
Interaction of sensitivity values with the calibration values for wearing position detection.

Touch sensor readings differ greatly between the states “correct/wrong position” and “glasses taken off” while only small differences are expected for the states “correct position” and “wrong position”. To take this into account, two parameters have been defined for the sensitivity of the touch sensors that can be individually configured: ambient sensitivity when glasses are taken off and wearing sensitivity when glasses are worn during individual calibration. These parameters increase the window in which a reading is considered valid in order to determine whether the glasses are worn in correct position or not (Fig 2).

If both of the temples’ readings are valid for each case, wearing or ambient, then such a result is recorded (“correct position” or “glasses taken off”), otherwise the state “wrong position” is recorded. It should be noted that a higher numeric value range increases the detection window but decreases the sensitivity.

#### Activity recognition

The activity recognition is based on a machine learning algorithm, which was developed and validated in a previous study [19]. Shortly, MATLAB R2017b (The MathWorks Inc., Natick, MA, USA) was used to develop the activity classifier, which processes and classifies the raw data from the three axes of the accelerometer into eight activity types (lieing, sitting, standing, walking, climbing stairs, running, jumping and cycling). The classifier is based on a cubic support vector machine (SVM) model and uses 13 time domain features to achieve an accuracy of about 93% while having a reduced memory size to enable execution on the low-power microcontroller. Moreover, we found that the activity classification match within the previous study protocol could be improved by applying an additional filter criterion.

**Fig 3:**
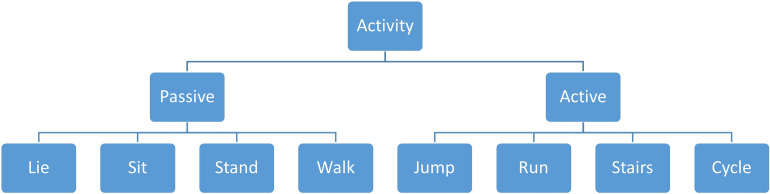
Hierarchical diagram of the activity classification.

The activity recognition is essential to realize the adaptive shutter operation, i.e. the occlusion rhythm for the healthy eye should be sensibly halted in situations with risk of injury or accident that would require the intermittent view of the healthy eye. For this purpose, we decided to split the eight activities in two groups–passive and active (motion)–to distinguish between movement-intensive activities such as jumping, running, climbing stairs, and cycling in contrast to non-intensive activities such as lieing, sitting, standing, and walking (Fig 3).

Once such active motion has been detected, this should halt the occlusion rhythm for the healthy eye until there is no active motion any longer. This in turn affects the calculation of the occlusion time, as the timer is halted in such situations.

#### Occlusion monitoring

The occlusion rhythm of the LCD lenses can be configured and depends on the therapy protocol. Also, the prescribed wearing and occlusion times can be set. The above mentioned integrated circuit TPS65735RSNR is controlled by the microcontroller firmware to perform the adaptive shutter operation. The daily therapy goal is achieved when the occlusion timer matches the prescribed occlusion time.

### Proof of concept study

In order to demonstrate the feasibility of continuous therapy monitoring with the first generation of miniaturized smart shutter glasses, we carried out a proof of concept study with healthy children. All parents gave informed and written consent and the nature of the study was explained to the children in an age-adapted fashion as well. The individual in this manuscript has given written informed consent (as outlined in PLOS consent form) to publish these case details. The study procedure was covered by approval by the local Ethics Committee (Ärztekammer Saarland, 73/18) and complied with the Declaration of Helsinki.

In total, seven healthy subjects were included in the study (4 females, 3 males, aged 2–9 years). The subjects were instructed to wear the assembled LCD shutter glasses in correct position and to perform the following activities: sitting, standing, walking, running, jumping, cycling, and climbing stairs according to a defined test protocol. Intentionally wearing the glasses in wrong position was not instructed to simplify the protocol for the children. The parents were instructed to document their child’s activities according to the test protocol that the subjects carried out in their everyday life, but under strict medical supervision and in the presence of their parents. In order to avoid an amblyogenic effect, the shutter cycle was set to 10 seconds occlusion on/30 seconds occlusion off, and a break was taken after 5 minutes at the latest. Special attention was also paid to the children during the activities, so that there was no danger for the children at any time. The activity was varied repeatedly and several times in accordance with the protocol specifications (note that lieing was not performed by the subjects although it is part of the trained activity classifier). All time intervals, the corresponding states and shutter status were recorded in handwriting by the same observer. A blinded person evaluated the event data analysis and compared it with the protocols.

## Results

### LCD shutter glasses for children

**Fig 4:**
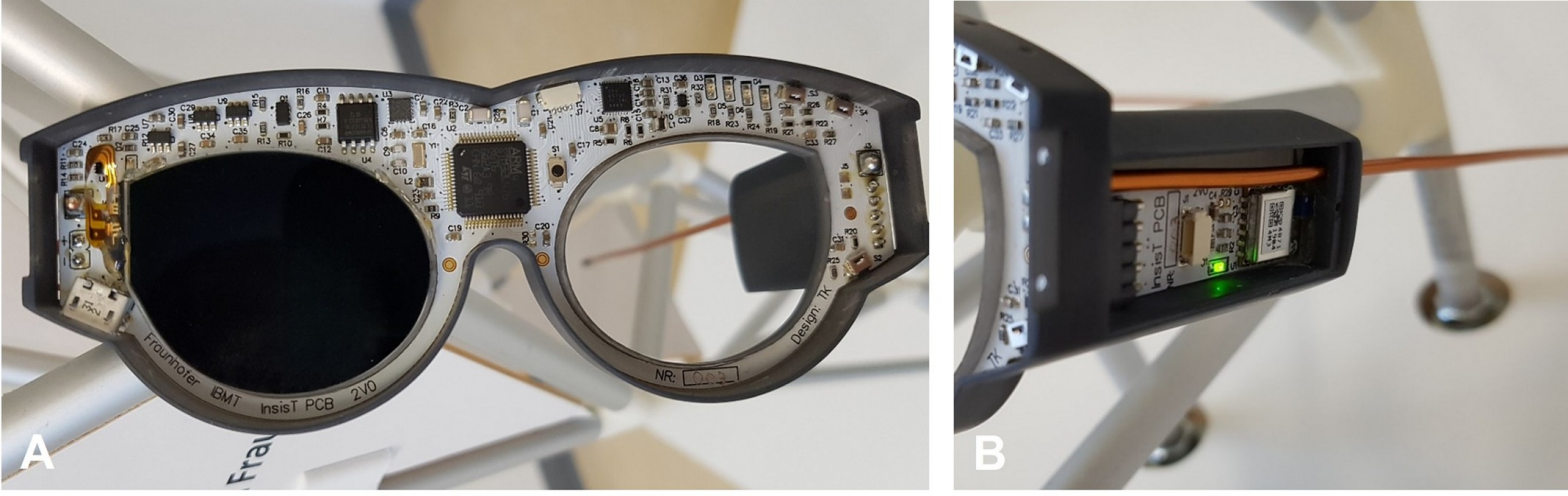
Photographs of electronic glasses frame. (A) Assembled LCD shutter glasses for children without frame cover and with one activated LCD lens. (B) Side view of the left temple with integrated Bluetooth module; the battery is located on the opposite side.

The miniaturized electronic glasses frame (Fig 4) was manufactured with very thin (0.6 mm) flexible FR4-based printed circuit board (Multi Leiterplatten GmbH, Brunnthal, Germany).

The main part (114.5 mm x 44 mm) has solder connections for the LCD lenses and for the temples and runs on a lithium-polymer rechargeable battery (3.7 V, 170 mAh). We used enameled copper wire (2 mm diameter) as temples because we achieved an improved sensing capability with it for the detection of the state of wearing position in our previous work [19]. In this study, the miniaturized electronic glasses frame performs therapy execution and data logging in order to proof the principles of monitoring wearing and occlusion times.

The assembled LCD shutter glasses for children have the following technical specifications:

- **Battery life** (@ 170 mAh)

Standby mode—approximately 20.5 days @ 340 μA

Therapy mode—approximately 26 hours @ 6.5 mA

- **Data logger** (@ 16 MB)

Recording of 1.78 million events, i.e. 123.5 days (@ a daily wearing time of 8 hours and storage of one event every 2 seconds)

- **Weight**

Approximately 45 g

### Therapy execution and monitoring system

Once the glasses have received therapy configuration, they execute the therapy protocol and monitor the progress of the applied therapy, considering the wearing position of the glasses, the type of activity performed by the wearer and the prescribed occlusion pattern. The therapy configuration data includes: device ID, therapy mode (standard or dynamic), wearing and occlusion times per day, occlusion pattern(s), occlusion lens (left or right).

To achieve such monitoring, the finite state machine shown in the S1 Fig has been designed. Please refer to this diagram to understand the detailed operation of the therapy monitor. As a simple overview, the following statements are true:

- Once the therapy configuration has been applied, a *daily_therapy_goal_achieved* flag is set to TRUE.
- The therapy is applied in the order from pattern 1 to pattern ‘n’ if more than one pattern is prescribed.
- If there is still wearing time left for that day after all patterns have been applied, therapy will continue without occlusion until the wearing time specified in the therapy configuration is reached.
- In the case of standard therapy mode, it depends on the determined wearing position whether the time for the applied therapy is accumulated or not.
- In the case of dynamic therapy mode, besides the wearing position, the activity of the wearer is also considered in order to accumulate (effective) time of therapy execution; i.e. if the therapy mode is dynamic and the classified activity is an active motion such as e.g. *jumping*, the time is not counted as therapy execution time.
- Once the daily therapy goal regarding the prescribed wearing and occlusion times is achieved, it is not possible to start a new therapy session.
- At the start of a new day or in case of a new therapy configuration, the *daily_therapy_goal_achieved* flag is cleared.

**Fig 5:**
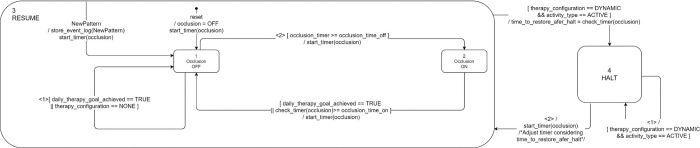
Occlusion finite state machine diagram.

In the context of therapy execution, the occlusion finite state machine is responsible for controlling the occlusion of the lenses. Occlusion depends on the therapy configuration currently being applied and, in case of the dynamic therapy mode, on the classified activity. As previously explained, once an active motion is detected, this will cause the occlusion timer to halt until a transition to a passive motion occurs. Fig 5 shows the operation of the occlusion finite state machine.

### Study outcome

Table 1 shows the averaged results for the precision of the state of wearing position detection and of the activity recognition. On average, there is a 72.6% agreement of the detected states of wearing position and an activity recognition match of 77.3%.

**Table 1:** Averaged results of the proof of concept study with healthy children.

Subject	State of wearing position [%]	Activity [%]
1	55.2	70.4
2	48.9	76.0
3	50.0	72.2
4	100.0	91.9
5	54.2	70.8
6	100.0	85.3
7	100.0	74.2
**Average**	**72.6**	**77.3**

**Fig 6:**
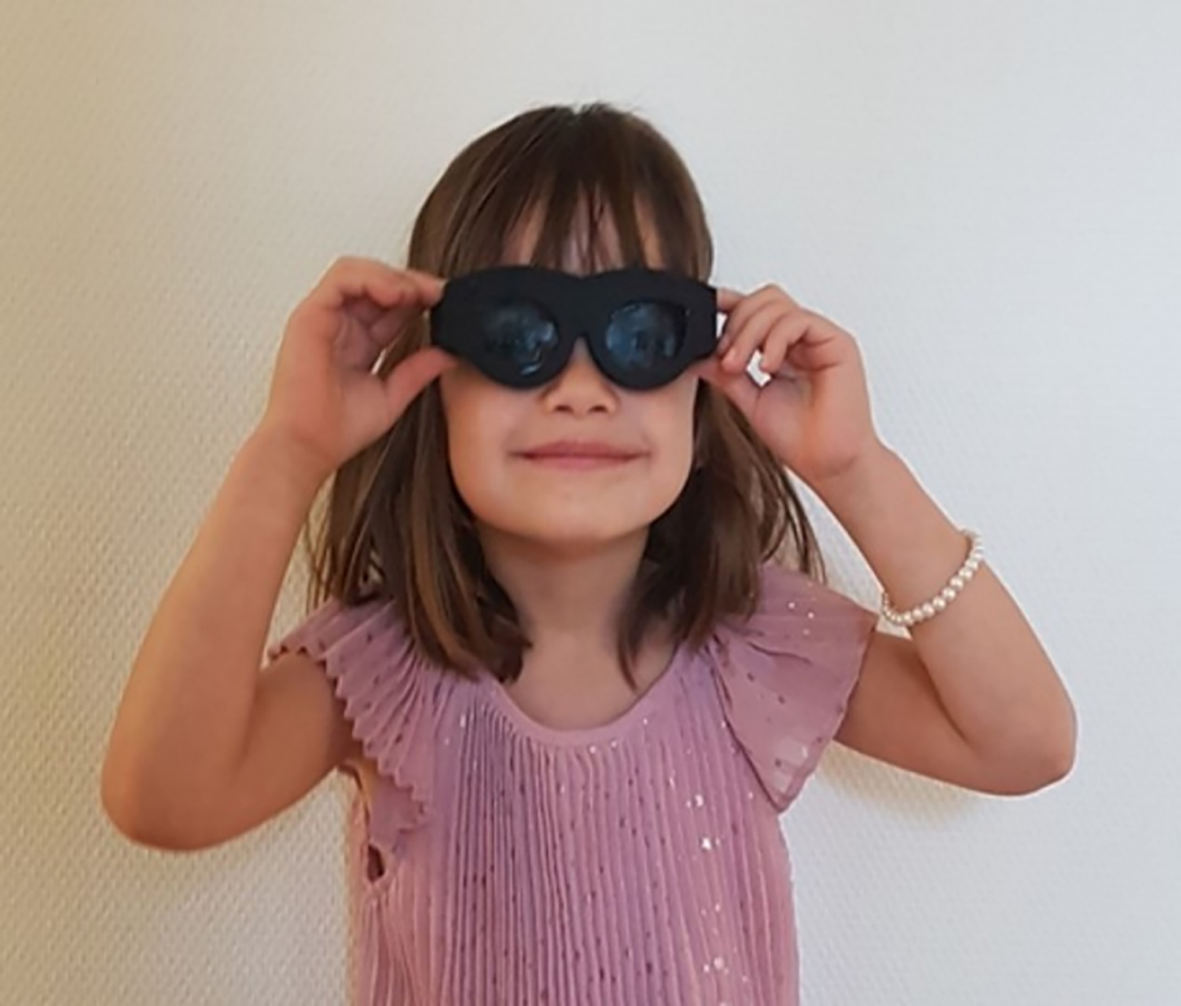
Happy child wearing the assembled LCD shutter glasses.

Removing the shutter glasses was always detected 100% correctly. In contrast to this, wearing the shutter glasses in correct position was only very good detected for subjects 4, 6 and 7. The other subjects suffered from an insufficient adaptation to the head shape, which is a known problem from our previous study with adults [19]. For technical developmental reasons, we only had one size of shutter glasses available, so that the fit could not be adapted to each individual child yet. The deterioration of the touch sensor’s sensitivity depending on the head shape and/or hairstyle was still there because the shutter glasses frame size, though much smaller than the previous one for adults, did not allow a perfect fit as close as possible to the skin for all children. This is reflected in the averaged results of the wearing position detection for subjects 1, 2, 3 and 5 (Tab. 1). The acceptance of the shutter glasses was nevertheless very good (Fig 6).

The individual activities were recognized with varying precision. For all subjects, the shutter glasses recognized sitting and standing very good whereas jumping and cycling were well recognized. In contrast to this, walking, running and climbing stairs were not well recognized for the subjects. Likewise, despite help from parents, climbing stairs and cycling as well as jumping were not well recognized in the 2-year-old subject due to the mobile development. However, if only subjects 4, 6 and 7 with a perfect fit of the shutter glasses are taken into consideration, the average activity recognition is better: sitting 100%, standing 94%, walking 48%, running 67%, cycling 63%, jumping 83%, and climbing stairs 67%. Overall, the adaptive shutter operation was halted 100% correctly in all subjects when active motion was detected.

**Fig 7:**
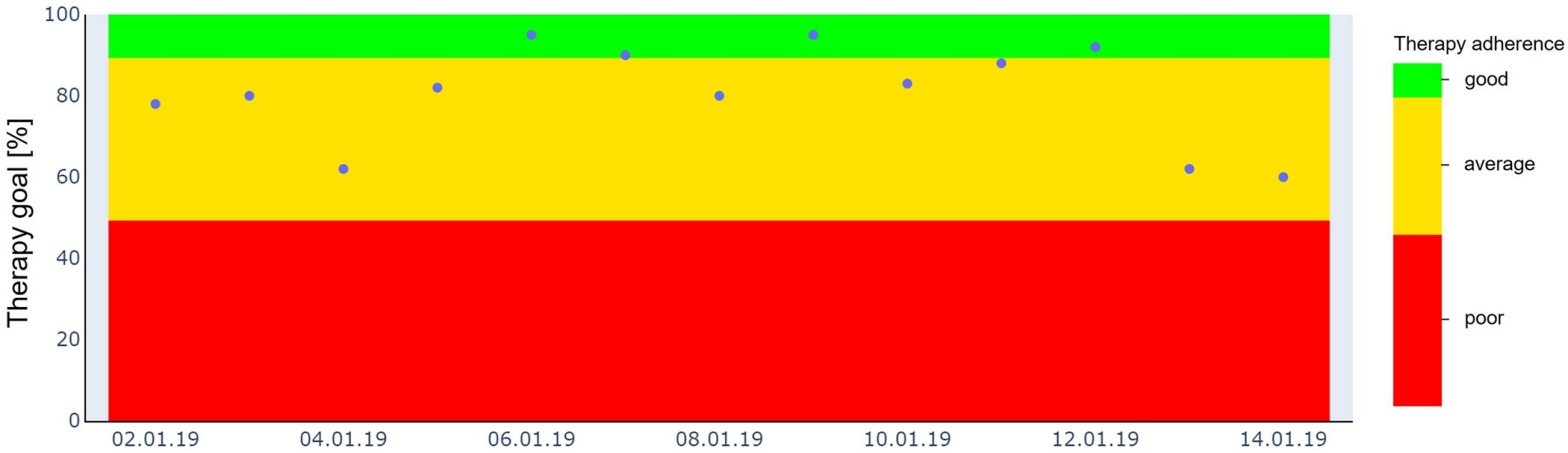
Example of a descriptive representation of the recorded data for the therapy adherence. Therapy adherence, i.e. the percentage of achieving the prescribed daily occlusion time, is split into three classes: good (90–100%, green), average (50–89%, yellow), and poor (0–49%, red).

Regarding therapy monitoring, Fig 7 shows an exemplary descriptive representation of recorded data for the continuous monitoring of the daily therapy goal (blue dots).

## Discussion

In this proof of concept study, we could show that our first generation of LCD shutter glasses with integrated sensors for children are suitable for demonstrating the feasibility of calculating wearing and occlusion times due to the development of smart algorithms for wearing position detection, activity recognition, and occlusion monitoring. This is an important step to improve therapy adherence in children and to reduce therapeutic burden for children and parents.

Our aim is to realize continuous therapy monitoring of children’s daily wearing and occlusion times in amblyopia treatment, thus enabling objective intervention studies. This therapy monitoring is actually mandatory, as the success of occlusion therapy depends on compliance, which is unsatisfactory [20–22], as shown in a study where patients wore the occlusion patch only 50% of the prescribed time [9]. By measuring the duration of wearing the glasses, the duration of effective occlusion and the state of wearing position, and thus determining actual compliance, treatment plans could be better adjusted through feedback to parents and physicians. This is of particular relevance as there is still a need for evidence-based treatment of amblyopia.

The actual problem with the objective measurement of compliance by using temperature sensors on patches so far is that the these sensors are rather big and difficult to handle [11–14]. Other disadvantages are the measurement inaccuracy at temperatures above 33°C [11, 12] and the lack of a wearing position control [14]. Furthermore, we were able to show in a previous study that integrated capacitive touch sensors are more reliable than temperature sensors [19].

The therapeutic use of a sensor-based glasses frame instead of sensor-enhanced patches also has the advantage that an additional refractive correction can be simultaneously integrated. Studies have already shown that LCD shutter glasses are a promising option for the treatment of amblyopia in children and may also be potentially less of a stigma than patches [15, 16, 18]. Furthermore, the presented instrumentation of shutter glasses with sensors and smart algorithms enables advanced features such as continuous therapy monitoring and personalized therapy, e.g. by recognizing the wearer’s activity and adapting the occlusion to the respective situation. In this way, the intermittent view of the healthy eye could be used in potentially dangerous situations. This could increase safety and adoption of LCD shutter glasses, potentially improving treatment adherence and ultimately efficacy. However, the weight of our assembled LCD shutter glasses is one aspect that should be further improved.

In addition, by integrating real-time multimodal sensing technology and processing power into LCD shutter glasses, feedback could be provided to both the parents and the patients when the glasses are worn incorrectly or removed. In this study, we could show that the wearing position can be successfully detected when the glasses had a perfect fit as it was in our previous study [19]. A correct wearing position is essential in therapy, as it would be possible to "wear" the glasses at the nose tip to not look through the glasses.

Even so, the real-time control algorithms detected the activity well in a proof of concept in children. We consider the recorded data to be reliable and the measurements of activity and wearing position to be robust. However, this was only a proof of concept study on a small number of subjects and due to the insufficient fit of the glasses, an exact measurement was not possible for all subjects. Nevertheless, due to the development progress, this is a satisfactory result and encourages continued work on the further development of this project.

In conclusion, we can summarize the following achievements and features of the assembled LCD shutter glasses:

- Electrically conductive temples as capacitive touch sensors and individual calibration routine for monitoring the wearing position
- Activity recognition and activity-dependent halt of the occlusion rhythm using classification of eight activities from acceleration data
- Continuous event recording for wearing position, activity and occlusion
- Continuous monitoring of wearing and occlusion times

**Fig 8:**
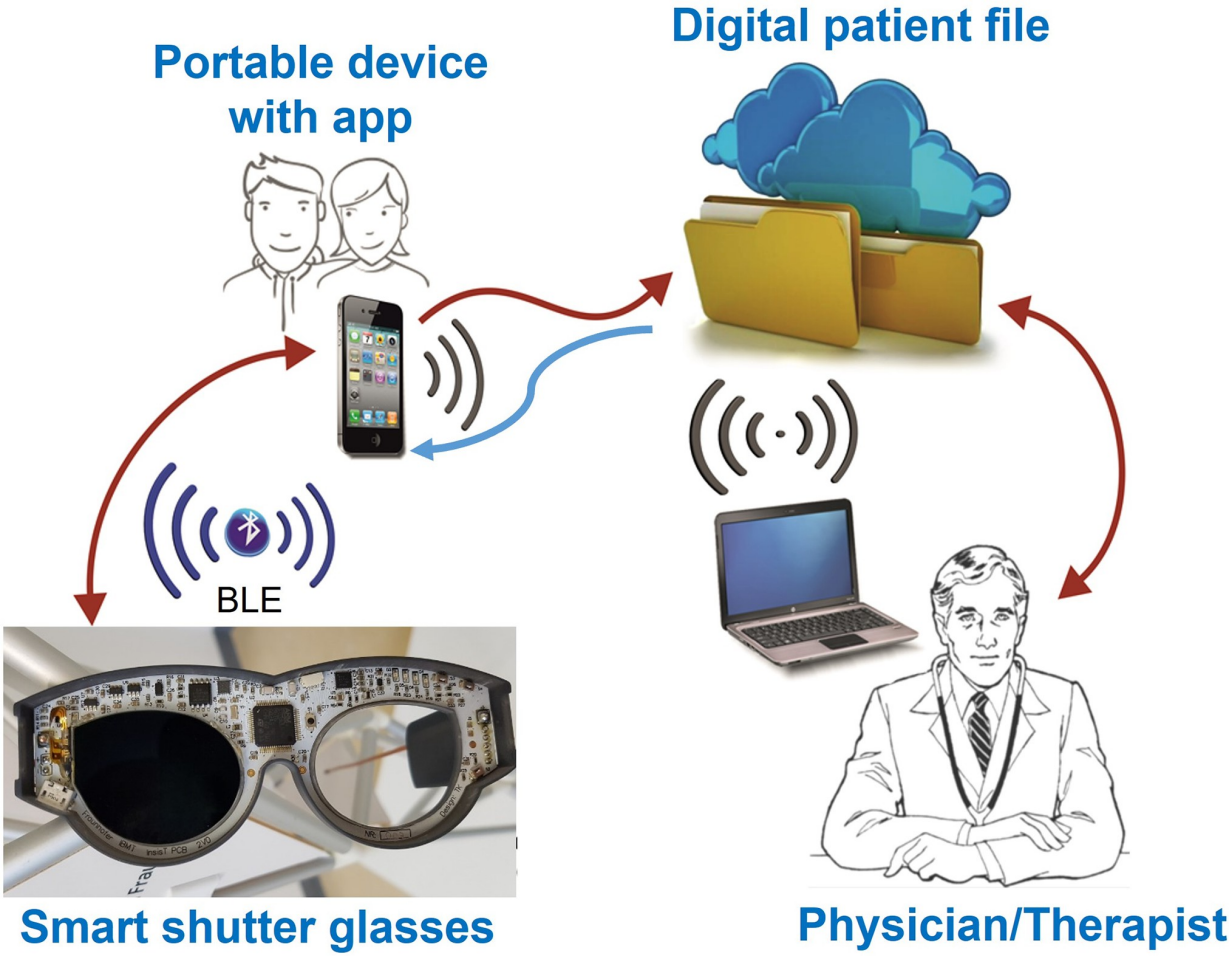
Illustration of the digital therapy system design. The smart shutter glasses, equipped with multimodal sensors and patient feedback, communicate bidirectionally with a smartphone via Bluetooth interface. The parents use the app for therapy monitoring. The smartphone securely transmits relevant data to a digital patient file accessible via the Internet, which in turn can be viewed by the attending physician/therapist.

Regarding future steps of research and development, such LCD shutter glasses could be part of a digital therapy system. Fig 8 shows an exemplary system design consisting of the smart shutter glasses to replace the unpleasant adhesive patch of classic occlusion therapy. In this therapy system, the parents are involved in therapy monitoring via a user-friendly app for smartphone and tablet. This app securely transmits the recorded therapy data to a digital patient file accessible via the Internet, which in turn can be used by the treating medical experts via a browser interface.

## Conclusion

The assembled smart shutter glasses for children are suitable for demonstrating the feasibility of continuous therapy monitoring by calculating wearing and occlusion times due to smart algorithms for wearing position detection, activity recognition, and occlusion monitoring. However, further research and studies are necessary to optimize the individual fit and performance of this wearable therapeutic device.

## Supporting information

S1 Fig Therapy monitor finite state machine diagram. (TIF)
