# Supplementary figures and images for: Monitoring of wearing and occlusion times with smart shutter glasses—A proof of concept
Source: PLoS One. 2022 Jun 24;17(6):e0270361. doi: 10.1371/journal.pone.0270361 (PMC9231704; doi:10.1371/journal.pone.0270361)

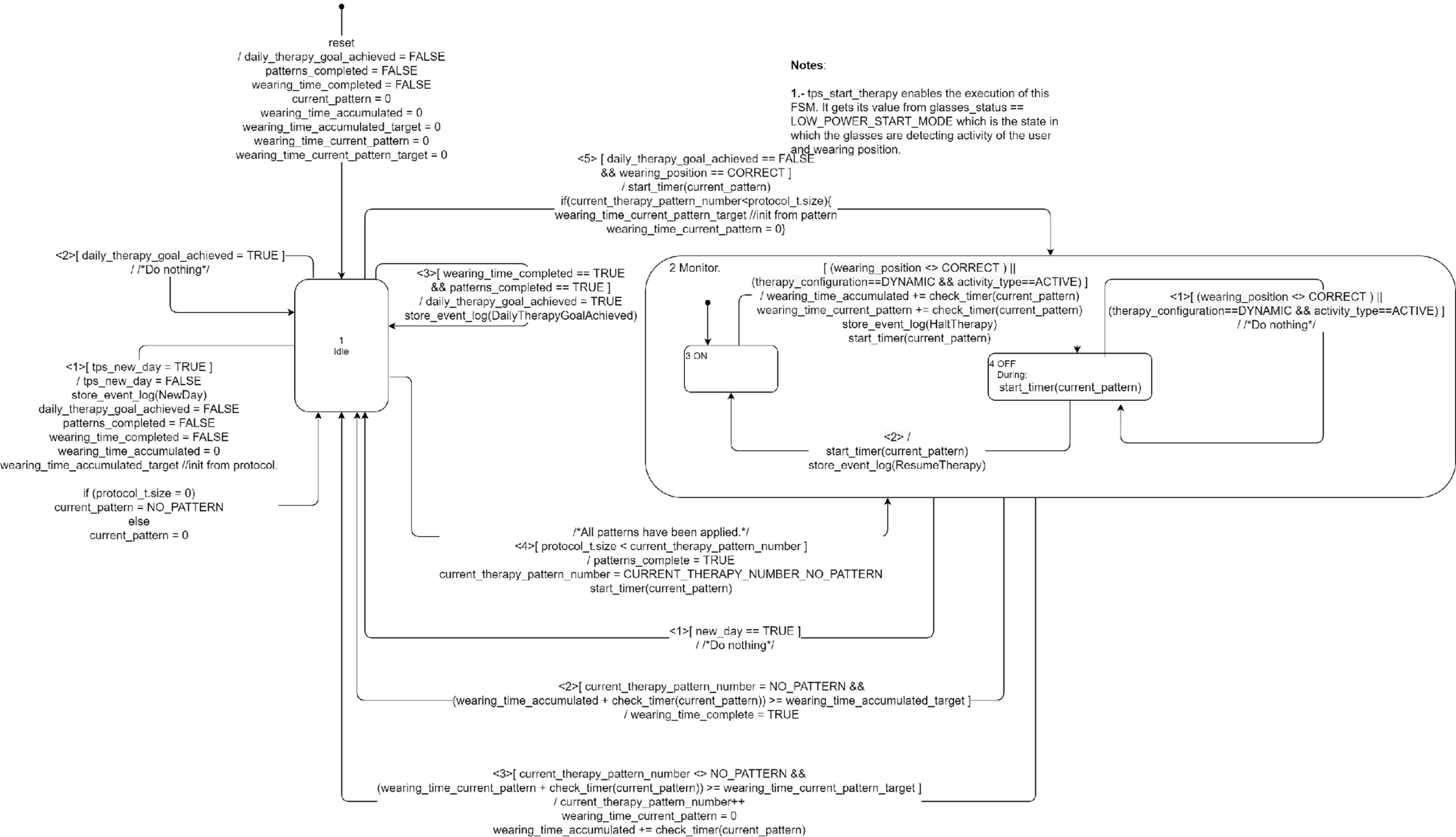

Supplement: S1 Fig — (TIF) [file pone.0270361.s001.tif]
